# Homocysteine: A Potential Biomarker for Diabetic Retinopathy

**DOI:** 10.3390/jcm8010121

**Published:** 2019-01-19

**Authors:** Amany Tawfik, Riyaz Mohamed, Nehal M. Elsherbiny, Margaret M. DeAngelis, Manuela Bartoli, Mohamed Al-Shabrawey

**Affiliations:** 1 Department of Oral Biology and Diagnostic Sciences, Dental College of Medicine, Augusta University, Augusta, GA 30912, USA; MALSHABRAWEY@augusta.edu; 2 Department of Cellular Biology and Anatomy, Medical College of Georgia (MCG), Augusta University, Augusta, GA 30912, USA; 3 Department of Ophthalmology, MCG, Augusta University, Augusta, GA 30912, USA; mbartoli@augusta.edu; 4 James and Jean Culver Vision Discovery Institute, MCG, Augusta University, Augusta, GA 30912, USA; 5 Department of Physiology and Endocrinology, MCG, Augusta University, Augusta, GA 30912, USA; rmohamed@augusta.edu; 6 Department of Biochemistry, Faculty of Pharmacy, Mansoura University, Mansoura 35516, Egypt; drnehal@hotmail.com; 7 Department of Ophthalmology, Department of Population Health Sciences, University of Utah School of Medicine, Department of Pharmacotherapy, School of Pharmacy, Salt Lake City, UT 84132, USA; margaret.deangelis@utah.edu

**Keywords:** diabetic retinopathy, homocysteine, biomarker

## Abstract

Diabetic retinopathy (DR) is the most common cause of blindness in people under the age of 65. Unfortunately, the current screening process for DR restricts the population that can be evaluated and the disease goes undetected until irreversible damage occurs. Herein, we aimed to evaluate homocysteine (Hcy) as a biomarker for DR screening. Hcy levels were measured by enzyme-linked immuno sorbent assay (ELISA) and immunolocalization methods in the serum, vitreous and retina of diabetic patients as well as in serum and retina of different animal models of DM representing type 1 diabetes (streptozotocin (STZ) mice, Akita mice and STZ rats) and db/db mice which exhibit features of human type 2 diabetes. Our results revealed increased Hcy levels in the serum, vitreous and retina of diabetic patients and experimental animal models of diabetes. Moreover, optical coherence tomography (OCT) and fluorescein angiography (FA) were used to evaluate the retinal changes in mice eyes after Hcy-intravitreal injection into normal wild-type (WT) and diabetic (STZ) mice. Hcy induced changes in mice retina which were aggravated under diabetic conditions. In conclusion, our data reported Hcy as a strong candidate for use as a biomarker in DR screening. Targeting the clearance of Hcy could also be a future therapeutic target for DR.

## 1. Introduction

Diabetic retinopathy (DR) is one of the most substantial microvascular complications of diabetes mellitus (DM), and is the most common cause of blindness in people under the age of 65 [1]. Unfortunately, DR can go undetected and not even noticed until irreversible damage and blindness has occurred [2]. DR is caused by damage to the blood vessels, resulting in retinal ischemia and increased permeability. New blood vessel formation (neovascularization) and diabetic macular edema (DME) are common characteristics for the disease [3]. Currently, retinopathy can only be diagnosed by a qualified specialist, either via direct proper examination of the eye or the examination of images captured by appropriate health care staff, which is costly and therefore restricts the population that can be effectively screened. An easily available, reliable screening biomarker of diabetic retinopathy would be of great benefit in identifying the population in need of further assessment and treatment [4]. Our research highlights homocysteine (Hcy) as a biomarker for DR that shows possibilities as a screening marker to detect early diabetic retinopathy or even to detect patients at an increased risk of DR at the time of diagnosis of diabetes. In addition, targeting Hcy clearance could be a future therapeutic target for DR.

Hcy is a sulfur-containing amino acid that is formed entirely upon the demethylation of the essential amino acid methionine, which is found principally in red meat and dairy products. Hcy is nutritionally controlled and metabolized through two pathways: The remethylation and trans-sulfuration pathways. Vitamins B_6_, B_12_ and folic acid (folate) serve as cofactors in Hcy metabolism. To accomplish normal metabolism of Hcy, the body requires adequate amounts of folic acid, vitamin B_12_ and vitamin B_6_, and due to the absence of these cofactors, Hcy can easily accumulate to harmful levels [5]. Normally, the human body is able to get rid of excess Hcy via the transsulfuration pathway, by which Hcy is converted to cystathionine by cystathionine β-synthase (CBS) enzyme by converting it into an antioxidant glutathione. The normal total plasma content of Hcy varies from 3–15 µM, and elevated plasma levels are termed hyperhomocysteinemia (HHcy). The ranges of Hcy elevated levels have been referred to as mild (16–30 µM), moderate (31–100 µM), or severe (>100 µM). High levels of Hcy in the blood have been reported to be an independent risk factor for heart disease as well as associated with kidney and brain disease [6,7,8].

Recently, elevated Hcy level has gained special consideration in relation to DR in several clinical studies, suggesting an association between elevated serum Hcy levels and the risk of DR [9,10,11,12,13,14,15,16,17]. There is an association between HHcy and diabetes-induced microangiopathies (diabetic nephropathy, retinopathy and macular edema) [18,19,20,21]. Studies suggested a strong relationship between elevated Hcy levels and DR. However, the exact role of HHcy in the development of DR is not clearly elucidated. There is clear evidence that Hcy induces the death of retinal ganglion cells in vitro [22] and in vivo [23]. Furthermore, vasculopathies linked to HHcy include endothelial dysfunction, vessel wall malformations, loss of extracellular matrix collagen, and disruption of the blood–brain barrier (BBB) in rodents and humans [24]. Impaired endothelial cell function has been also reported in vitro and in vivo in HHcy [25]. Moreover, our previous work reported a direct impact of excess Hcy on the blood–retinal barrier (BRB), induced retinal ischemia and neovascularization, increased vascular endothelial growth factor (VEGF) level in retina [26,27,28], activation of endoplasmic reticulum (ER) stress [29], activation of oxidative stress [30] and induced epigenetic modifications [31].

## 2. Experimental Section

### 2.1. Animals

All animal procedures followed the Association for Research in Vision and Ophthalmology (ARVO) Statement for Use of Animals in Ophthalmic and Vision Research policies and were accomplished in accordance with the Institute for Animal Care and Use Committee and Augusta University policies (IACUC Approval for Protocol 2014-0683) C57Black6 mice, Akita mice and Sprague–Dawley (SD) rats were obtained from Jackson Laboratories). Type 2 diabetic mice (db/db) were also obtained from Jackson Laboratories and bred according to the Jackson Laboratory recommendations (BKS.Cg-*Dock7^m^+/+Lepr^db^*/J). All animals were group-housed, subjected to the standard 12-hour light/12-hour dark cycle, provided with food and water ad libitum and kept at a temperature range of 22–24 °C. Genotypes were confirmed according to the Jackson Animal Laboratory genotyping protocols specific to each genotype.

### 2.2. STZ Injections

Six to eight-week old C57BL/6 mice and Sprague–Dawley (SD) rats were injected intraperitoneally (IP) with streptozotocin (STZ) at 50 mg/kg to induce diabetes. Mice were injected with STZ for three consecutive days, while SD rats received a single IP dose (animals were fasted for 4 h prior to injection). The mice and rats were evaluated 3 days after the STZ injection series to determine if they were sufficiently diabetic. A blood glucose concentration that exceeded 300 mg/dL was considered diabetic.

### 2.3. Measurement of Homocysteine Level

The concentration of Hcy in the blood and retinas of humans and different animal models of diabetes, representing type 1 and type 2 diabetes (STZ-treated mice, db/db mice and STZ-treated rats)—was determined using an Hcy Enzyme Linked Immunosorbent Assay (ELISA) kit from Cell Bio Labs Inc (STA-670) (San Diego, CA, USA). Human vitreous samples were a generous gift from Dr. Gregory I. Liou, Department of Ophthalmology, and Augusta University. Samples were derived from donors (Georgia Eye Bank, Atlanta, GA, USA). The selection criteria included an age >50 years old, with donors being either insulin-requiring diabetes (with DR) or normal control and no life-support measures. The eyes were enucleated an average of 6.71 ± 0.84 h after death. Postmortem eye specimens were utilized for vitreous collection by aspiration [32]. The patient’s blood samples were provided by Dr. Margaret M. DeAngelis and IRB was approved by the University of Utah. The collected retinas were rinsed with 1×phosphate-buffered saline (PBS), homogenized in 1× PBS and kept at −20 °C overnight. To break the cell membranes, two freeze–thaw cycles were performed, and then the homogenates were centrifuged for 5 min at 5000× *g* at 4°C. The supernatant was separated and assayed immediately according to the protocol provided with the kit. The blood samples collected were allowed to clot in serum separator tubes (SST) for a minimum of 2 h at room temperature prior to centrifugation at 1000× *g* for 15 min. The serum was collected and immediately assayed according to the protocol provided with the kit. The readings were taken at 450 nm using an ELISA plate reader.

### 2.4. Measurement of Cystathionine Beta-Synthase(CBS)Enzyme Level

The concentration of CBS in the serum from human patients with and without diabetes and from diabetic mice was determined using a CBS ELISA assay kit from My BioSource (MBS700623) (San Diego, CA, USA). Blood samples were allowed to clot in serum separator tubes (SST) for a minimum of 2 h at room temperature prior to centrifugation at 1000× *g* for 15 min. The serum was collected and immediately assayed according to the protocol provided with the kit.

### 2.5. Optical Coherence Tomography (OCT) and Fluorescein Angiography (FA)

OCT and FA imaging were used to evaluate the retinal vasculature in living mice according to our published methods [28,31]. Briefly, 2% isoflurane was used to anesthetize mice and 1% tropicamide eye drop was used to dilate their pupils. The anesthetized mice were then individually placed on the imaging platform of the Phoenix Micron III retinal imaging microscope accompanied with an OCT imaging device (Phoenix Research Laboratories, Pleasanton, CA, USA). Then, Genteal gel was applied liberally to keep the eye moist during imaging. For FA, 10% fluorescein sodium (Apollo Ophthalmics, Newport Beach, CA, USA) was injected to the mice (IP, 10 to 20 µL), and fluorescent images were rapidly captured for ~5 min. Indistinct vascular borders progressing to diffusely hazy fluorescence was considered as fluorescein leakage.

### 2.6. Immunofluorescent Assessment of Hcy Level

Retinal cryosections were prepared according to our previously published method [28,30]. First, the retinal sections were fixed with 4% paraformaldehyde. The fixation was followed by washing with PBS–Triton X-100, blocking with Power Block (BioGenex, Fremont, CA, USA), and then incubation with an anti-homocysteine antibody (Catalog number; ab5512 rabbit polyclonal, Chemicon International Inc., Temecula, CA, USA) at 37 °C for 3 h or at 4 °C overnight. The sections were subsequently washed with PBS–Triton X-100 three times. Then, the sections were incubated with the appropriate secondary antibody at 37 °C for 1 h. Sections were then washed with PBS–Triton X-100 and Fluoroshield with DAPI (4’6-diamidino-2-phenylindole) was applied, followed by the placement of a coverslip (Sigma Aldrich F6057, Saint Louis, MO, USA) to label the nuclei. Thereafter, sections were examined and images were captured using fluorescent microscopy (Carl Zeiss, Göttingen, Germany).

### 2.7. Statistical Analysis

The results are expressed as mean ± standard deviation SD. The assessment of differences among experimental groups was performed using the two-tailed *t* test or one-way analysis of variance (ANOVA). Detection of statistical differences by ANOVA was followed by a post hoc Tukey’s test to determine which groups differed. Statistical significance was considered at a confidence level of *p* < 0.05.

## 3. Results

### 3.1. Elevated Hcy Levels in Serum, Vitreous and Retina of Diabetic Patients and Experimental Diabetic Animals

**Figure 1:**
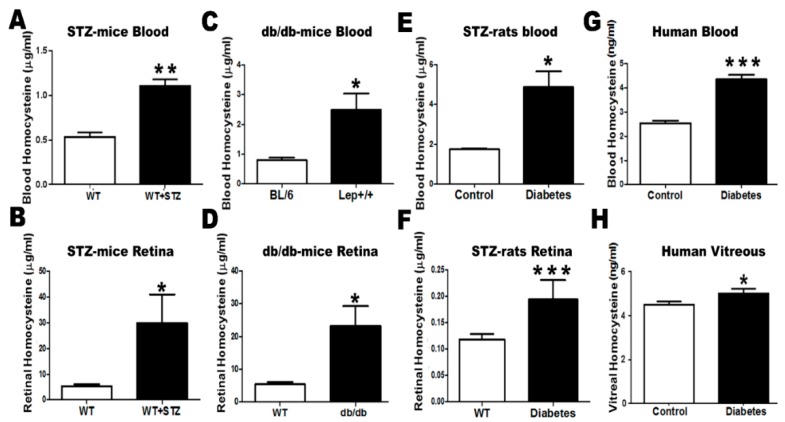
Assessment of Hcy level by enzyme-linked immuno sorbent assay (ELISA). (**A**) Blood and (**B**) retina of streptozotocin (STZ)-injected mice showed a significant increase in Hcy level compared to the wild-type (WT) group (* *p* < 0.05, ** *p* < 0.01). (**C**) Blood and (**D**) retina of genetically obese leptin receptor-deficient mice (db/db) showed a significant increase in Hcy level compared to the WT group (* *p* < 0.05). (**E**) Blood and (**F**) retina of STZ-injected rats showed a significant increase in Hcy level compared to the WT group (* *p* < 0.05, *** *p* < 0.001). (**G**) Blood and (**H**) vitreous of diabetic patients showed a significant increase in Hcy level compared to the control non-diabetic group (*** *p* < 0.001, * *p* < 0.05).

To evaluate Hcy as a potential biomarker for the development of retinal complications in diabetes, we tested changes in Hcy level in the blood and retina of experimental models of type1and type 2 diabetes (STZ mice and rats and db/db mice, respectively). Our results showed increased Hcy levels in the blood and retina of diabetic animals compared to corresponding non-diabetic controls. In humans, the serum and vitreous levels of Hcy were significantly increased in diabetic patients compared to non-diabetic controls, suggesting that elevated Hcy levels might represent a risk factor for development of DR **(**Figure 1).

### 3.2. Hcy Immunolocalization in the Retina of Diabetic Patients and Experimental Diabetic Animals

**Figure 2:**
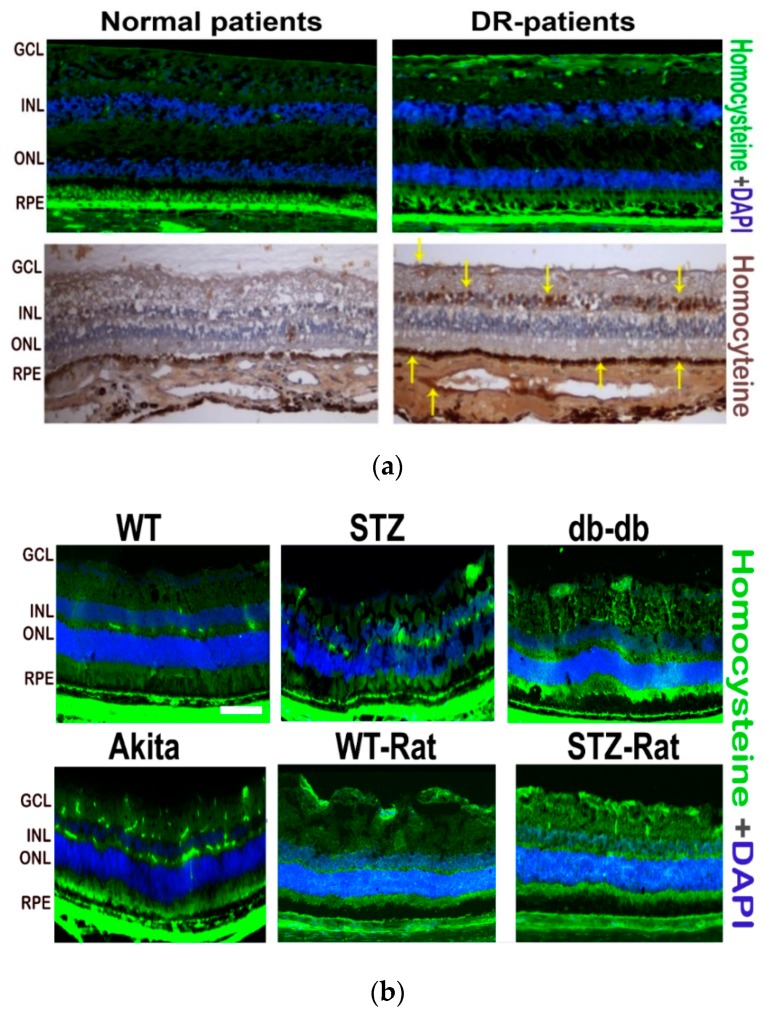
Immunolocalization of Hcy in diabetic retina. (**a**) Immunoflourescent staining (top panel) of retinal cryosections from diabetic patients (green fluorescence detects Hcy and 4’6-diamidino-2-phenylindole (DAPI) (blue) detects nuclei). Immunohistochemical detection (bottom panel) of Hcy (brown) in retinal cryosections from diabetic patients (yellow arrows). (**b**) Immunoflourescent staining of retinal cryosections from wild-type (WT) mice (upper left panel), STZ-injected mice (upper middle panel), db/db mice (upper right panel), Akita mice (lower left panel), WT rats (lower middle panel) and STZ-injected rats (lower right panel). Green fluorescence detects Hcy and DAPI (blue) detects nuclei.

In order to identify Hcy expression in the retina of diabetic patients, we performed Hcy immunostaining in frozen sections from human diabetic retina. Our results showed both marked immunoflourescent and immunohistochemical staining of Hcy in diabetic human retinal sections (Figure 2a). Further, we performed Hcy immunofluorescence in various experimental models of type1and type 2 diabetes. STZ-injected mice and rats as well as Akita mice were used as experimental models of type 1 diabetes. However, db/db mice were used as an experimental model of type 2 diabetes. Immunoflourescent staining showed an increased expression of Hcy in retinal sections from various diabetic animals compared to the corresponding WT non-diabetic controls (Figure 2b).

### 3.3. Down Regulation of CBS in the Serum of Diabetic Patients and Experimental Diabetic Animals

**Figure 3:**
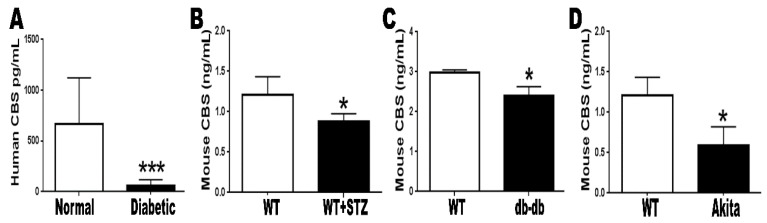
Assessment of cystathionine beta-synthase (CBS) level by ELISA. (**A**) Serum samples from diabetic patients demonstrated a significant decrease in CBS level compared to the control non-diabetic group (*** *p* < 0.001). (**B**) STZ-injected mice, (**C**) db/db mice and (**D**) Akita mice demonstrated a significant decrease in blood CBS level compared to corresponding control groups (* *p* < 0.05).

Cystathionine beta-synthase (CBS) catalyzes the first step in Hcy clearance through the transsulfuration pathway by converting Hcy and serine to cystathionine and water. To determine whether changes in CBS levels are associated with altered Hcy levels in diabetes, we tested the CBS level in the serum of diabetic patients and experimental diabetic animals. Our data demonstrated significantly lower levels of CBS in diabetic subjects compared to non-diabetic control (Figure 3).

### 3.4. Optical Coherence Tomography (OCT) and Fluorescein Angiography (FA)

**Figure 4:**
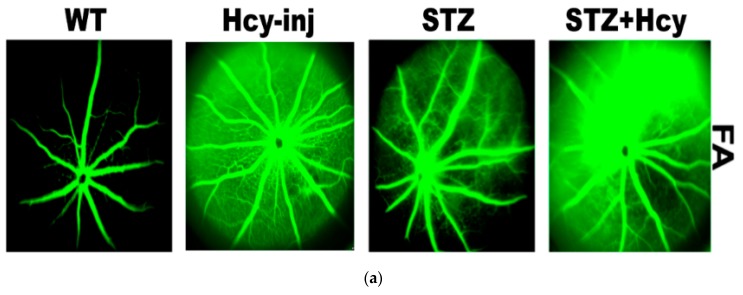

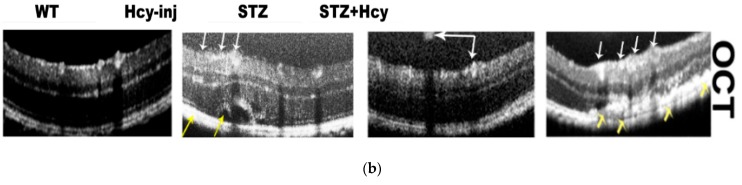
Retinal Fluorescein Angiography (FA) and Optical Coherence Tomography (OCT) assessment of wild-type (WT) mice, Hcy-intravitreal injected mice, STZ-injected mice and Hcy+STZ injected mice (8–10 weeks diabetic). FA demonstrated well-formed vessels in WT mice. FA for Hcy-injected mice showed hyperfluorescence, indicating vascular leakage. Similarly, the FA of STZ-injected mice showed decreased vessel integrity and a disrupted blood–retinal barrier (BRB), indicated by increased fluorescein leakage (pale green haziness). However, the FA of Hcy injected STZ- mice showed focal spots of hyperfluorescence, indicating more significant vascular leakage (**A**). OCT examination showed a typical normal architecture of retinal layers in WT mice, but a disruption of retinal morphology in Hcy-injected mice (white and yellow arrows). These changes were also observed in the OCT of STZ-injected mice. However, the OCT of Hcy+STZ injected mice demonstrated marked structural alteration with sub-retinal fluid leakage and neovascularization in both the inner retina (white arrows) and outer retina (yellow arrows) (**B**).

To confirm the link between Hcy and microvascular dysfunction in diabetic retina, OCT and FA were performed in WT and diabetic mice injected intravitreally with Hcy. Consistent with our previous publications [26,27,28,31], in the current study, OCT examination showed alterations in retinal vasculature with neovascularization in both inner (white arrows) and outer retina (yellow arrows), and disrupted retinal morphology, such as sub-retinal fluid accumulation, separation of the retinal pigmented epithelial layer, and thickening of the basal laminar membrane and the choroid, suggesting neovascularization. Also, STZ-injected mice demonstrated decreased vessel integrity and an impaired blood–retinal barrier (BRB), indicated by increased fluorescein leakage as well as disrupted retinal morphology, when compared to the WT mice. Furthermore, to evaluate the changes of the combined effect of elevated Hcy and diabetes on the retina, STZ-treated mice given intravitreal injections of Hcy were subjected to FA and OCT. Hcy-injected diabetic mice showed more deleterious effects on the retinal architecture, decreased vessel integrity and more impairment of the BRB.FA showed increased fluorescein leakage with focal spots of hyperfluorescence, and OCT results showed more distortion in retinal morphology and BRB integrity and neovascularization compared to Hcy-injected and diabetic mice alone (Figure 4).

## 4. Discussion

This study was conducted to highlight Hcy as a marker and may be a future therapeutic target for DR. Recently, the association between Hcy and (DM) has gained increasing attention. Many studies reported increased levels of Hcy in the plasma of type 1 diabetes mellitus (T1DM) patients compared to non-diabetic controls [33,34]. It was also reported in a meta-analysis study that plasma Hcy concentrations in T1DM patients without any complications were normal compared with healthy people. However, plasma Hcy concentrations showed significant elevations only in T1DM patients with DR compared with T1DM patients without any complications [12], suggesting that increased Hcy levels during DM contributes to the development of retinopathy. In contrast, some studies reported no significant difference in Hcy levels between T1DM and non-diabetic patients [35], yet patients with proliferative retinopathy display significantly higher values of Hcy than those without [21]. Hcy level was also reported to be more elevated in type 2 diabetes mellitus (T2 DM) patients than controls [36,37,38,39,40]. On the other hand, some studies refuted the link between HHcy and incidence of retinopathy in diabetic patients [40,41]. Indeed, it was reported that T1DM patients had lower levels of Hcy and global DNA methylation [42]. A recent study reported that elevated Hcy levels were associated with an increased risk of DR, especially in T2DM patients [43].

Therefore, the current study aimed to solve this controversy by confirming what has been reported in human studies and also measured Hcy levels in different animal models of DM representing T1DM (STZ mice, Akita mice and STZ rats) and T2 DM (db/db mice). Our study showed a significant increase in Hcy levels in both human and different animal models of diabetes. There are many causes that have been suggested for increased Hcy levels, such as the deficiency of vitamin cofactors needed for Hcy metabolism, such as folic acid and vitamin B_12_ [44,45,46,47], or the deficiency of any of the enzymes involved the remethylation [48] or transsulfuration [49] pathways of the Hcy metabolism. Our study showed that the differences in the serum and retinal levels of Hcy were statistically significant between the control and diabetic groups in both humans and animals. In addition, our study showed a significant reduction of the CBS enzyme level, which is a key enzyme needed for Hcy clearance via the transsulfuration pathway, in both human and animal models of DR. Ratnam et al.reported that insulin plays a role in regulating Hcy metabolism, and impaired insulin levels in diseases such as diabetes may influence Hcy metabolism by regulating the hepatic transsulfuration pathway [50]. Diabetic nephropathy is a common microvascular complication of diabetes and could play a role in elevated Hcy levels due to chronic kidney insufficiency and impaired Hcy clearance. However, a recent clinical study was conducted on 163 normo-albuminuric patients with T1DM and normal renal function to examine whether there is an independent relationship between plasma total homocysteine (tHcy) and retinopathy in normo-albuminuric T1DM patients with normal estimated glomerular filtration rate (eGFR). The study suggested that tHcy is independently associated with retinopathy in normo-albuminuric T1DM with normal eGFR [17]. Furthermore, another large study of European type 1 diabetic patients stated that increased concentrations of tHcy were independently related to macro-albuminuria, renal function and hypertension [51].

Moreover, various studies reported the mechanism of action of Hcy leading to retinal neurodegeneration using different animal models. Hcy has been reported to induce apoptosis in retinal ganglion cells and induced ganglion cell loss via the dysregulation of mitochondrial dynamics in vivo and in vitro [52,53,54]. The activation of N-methyl D-aspartate (NMDA) receptors has been also suggested as a possible mechanism of HHcy-induced retinal ganglion cell death during DR in several studies [54,55,56,57,58,59]. Other studies suggested that HHcy exerts its toxic effect via the activation of inflammatory and oxidative stress mechanisms leading to the activation of mitogen-activated protein kinases (MAPK), macrophage infiltration and enhanced pro-inflammatory cytokines production [60]. Moreover, HHcy elicits oxidative stress and decreases nitric oxide’s bioactivity, leading ultimately to vascular dysfunction [61]. Our previous work demonstrated that HHcy caused dysfunction of the BRB, disrupts retinal pigment epithelial structure and function, activates oxidative and endoplasmic reticulum stresses and induces retinal neovascularization and epigenetic modifications [27,28,29,30,31]. The current study found that HHcy caused similar structural changes to what has been reported in our previous publications [27,28,31] when injected intravitreally in mice retina, and these changes were more deleterious when Hcy was injected in diabetic mice, suggesting the involvement of Hcy in the pathogenesis of diabetes-induced retinal damage. This was consistent with what has been reported by Srivastav et al.—That Hcy level was correlated with the decrease in the thickness of the retinal nerve fiber layer in diabetic patients—and suggested a correlation between increased serum levels of Hcy and an increased severity of retinopathy [11].

## 5. Conclusions

The current study is innovative in suggesting a correlation between elevated levels of Hcy in serum and in retina and DR in both diabetic human and animal models of diabetes. Furthermore, our results suggest an association between increased serum levels of Hcy and an increased severity of retinopathy. Therefore, Hcy could be a useful diagnostic marker for screening to predict the incidence and severity of retinal damage in diabetic patients. In addition, enhancing Hcy clearance via pharmacological or genetic manipulations could be a future preventive/therapeutic strategy in targeting diabetic retinopathy.
